# mmu-miR-374b-5p modulated inflammatory factors via downregulation of C/EBP β/NF-κB signaling in Kupffer cells during *Echinococcus multilocularis* infection

**DOI:** 10.1186/s13071-024-06238-0

**Published:** 2024-03-29

**Authors:** Guiting Pu, Yanping Li, Tingli Liu, Hong Li, Liqun Wang, Guoliang Chen, Shanling Cao, Hong Yin, Tharheer Oluwashola Amuda, Xiaola Guo, Xuenong Luo

**Affiliations:** 1 State Key Laboratory for Animal Disease Control and Prevention, Key Laboratory of Veterinary Parasitology of Gansu Province, Lanzhou Veterinary Research Institute, Chinese Academy of Agricultural Sciences (CAAS), Lanzhou, 730046 Gansu Province People’s Republic of China; 2 Jiangsu Co-Innovation Center for the Prevention and Control of Important Animal Infectious Disease and Zoonoses, Yangzhou University, Yangzhou, 225009 People’s Republic of China

**Keywords:** *Echinococcus multilocularis*, miR-374b-5p, Inflammatory factors, C/EBP β, NF-κB

## Abstract

**Background:**

Alveolar echinococcosis (AE) is an important infectious disease caused by the metacestode larvae of *Echinococcus multilocularis*, seriously threatening global public health security. Kupffer cells (KCs) play important roles in liver inflammatory response. However, their role in hepatic alveolar echinococcosis has not yet been fully elucidated.

**Methods:**

In this study, qRT-PCR was used to detect the expression level of miR-374b-5p in KCs. The target gene of miR-374b-5p was identified through luciferase reporter assays and loss of function and gains. Critical genes involved in NFκB signaling pathway were analyzed by qRT-PCR and western blot.

**Results:**

This study reported that miR-374b-5p was significantly upregulated in KCs during *E. multilocularis* infection and further showed that miR-374b-5p was able to bind to the 3'-UTR of the *C/EBP β* gene and suppressed its expression. The expression levels of NF-κBp65, p-NF-κBp65 and pro-inflammatory factors including iNOS, TNFα and IL6 were attenuated after overexpression of miR-374b-5p while enhanced after suppression of miR-374b-5p. However, the Arg1 expression level was promoted after overexpression of miR-374b-5p while suppressed after downregulation of miR-374b-5p. Additionally, increased protein levels of NF-κBp65 and p-NF-κBp65 were found in the C/EBP β-overexpressed KCs.

**Conclusions:**

These results demonstrated that miR-374b-5p probably regulated the expression of inflammatory factors via C/EBP β/NF-κB signaling. This finding is helpful to explore the mechanism of inflammation regulation during *E. multilocularis* infection.

**Graphical Abstract:**

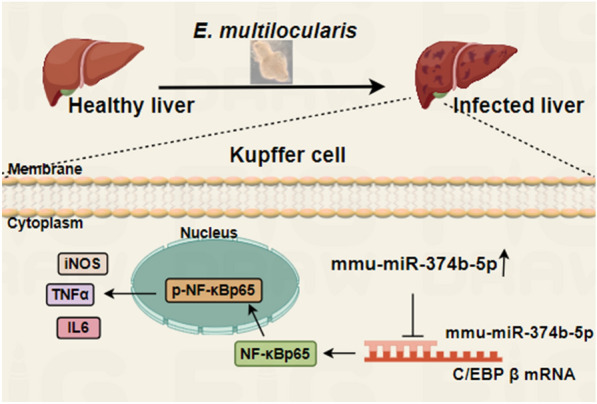

**Supplementary Information:**

The online version contains supplementary material available at 10.1186/s13071-024-06238-0.

## Background

In recent studies, increasing attention has been given to zoonotic alveolar echinococcosis (AE), which is caused by the larvae of *Echinococcus multilocularis* [1]. It is prevalent in areas with developed animal husbandry [2, 3]. The life cycle of *E. multilocularis* mainly involves rodents, foxes and humans. Humans have occasionally been infected by ingesting food or drinking water contaminated with eggs discharged from the definitive host foxes and dogs [4]. Once ingested, the oncospheres hatch from the eggs and then migrate from blood to body, especially liver. Remarkably, *E. multilocularis* mainly destroys the host’s liver by mechanical extrusion and production of active molecules [5]. Kupffer cells (KCs) play important roles in maintaining immune homeostasis of the liver against pathogen infection [6, 7]. KCs mediate liver injury and plerosis and contribute to formation of liver fibrosis by secreting cytokines, phagocytosis and antigen presentation [8, 9]. KCs can produce inflammatory cytokines or chemokines, which result in liver fibrosis during *Schistosoma japonicum* infection [10]. However, in the *E. multilocularis*-infected liver, the regulation of host immunity by KCs has not been clarified.

MicroRNAs (miRNAs), with 18–25 nucleotides, are a class of conserved endogenous non-coding RNAs with gene regulatory functions. miRNAs can silence target gene expression by base pairing to a complementary sequence in the 3ʹ-untranslated region [11]. miRNAs have been reported to participate in host-pathogen interactions. Recent studies have confirmed that miRNAs can play a key role in the regulation of inflammatory responses against pathogens [12]. In the T helper cells, schistosomal miR-10 targets MAP3K7 and consequently downmodulates NF-κB activity [13]. *Leishmania braziliensis* exploits host miR-548d-3p to modulate the production of pro-inflammatory cytokines, affecting inflammatory processes [14]. Therefore, study of miRNAs will provide further insight into parasite-host interactions [15, 16].

Previous studies found that miR-374b-5p was involved in different physiological and pathological processes [17–19]. In this study, we found that mmu-miR-374b-5p (miR-374b-5p) was significantly upregulated in the KCs of mice infected with *E. multilocularis*. Therefore, we hypothesized that miR-374b-5p might mediate the inflammatory response in mouse liver during *E. multilocularis* infection. This finding will demonstrate a function of miR-374b-5p in regulation of KC immune responses, possibly involved in the host-parasite relationship during *E. multilocularis* infection.

## Methods

### Parasite infection

Fresh protoscoleces (PSCs) were aseptically dissected from *E. multilocularis*-infected gerbils. The activity and quantity of protoscoleces were determined by microscopic observation. Six-week-old female Balb/c mice (*n* = 80) were purchased from Laboratory Animal Center of Lanzhou Veterinary Research Institute and were randomly allocated into two groups of 40 each. One group was intra-peritoneally inoculated with approximately 1000 protoscoleces, while the control group (C) was treated with the same volume of PBS at the same time [20].

### Collection of excretory/secretory products (ESPs)

*Echinococcus multilocularis* were cultured in DMEM medium with 1% penicillin-streptomycin (without FBS) in an incubator containing 5% CO_2_ at 37 ℃. After 8 h, the culture medium was removed, and fresh culture medium (without FBS) was added. ESPs of protoscoleces were obtained at 24 h. Then, the protein concentration was determined by BCA kit (Vazyme, China). The endotoxin in ESPs was removed using Pierce High-Capacity Endotoxin Removal Resin (Thermo, USA), and the amount of endotoxin was determined by the ToxinSensor^**™**^ Chromogenic LAL Endotoxin Assay Kit (GenScript, USA). The amount of endotoxin concentration was < 0.05 EU/ml.

### Isolation, cultivation and transfection of KCs

Primary KCs were isolated from the uninfected (*n* = 3) and *E. multilocularis*-infected (*n* = 3) mouse liver by collagenase perfusion method and density gradient centrifugation [21]; then, they were cultured in RPMI-1640 medium supplemented with 10% FBS and 1% penicillin-streptomycin. Four hours later, the medium was removed, and adherent cells were cultured continually with fresh RPMI-1640 medium. The cells were maintained in an incubator containing 5% CO_2_ at 37 ℃.

KCs were plated into 12-well plates and transfected with 100 nmol/l miR-374b-5p mimics, 100 nmol/l miR-374b-5p inhibitor or 100 nmol/l of their NC (negative control, Sangon China) using Lipofectamine^™^ RNAiMAX Transfection Reagent (Invitrogen, USA), respectively. Following transfection for 10 h, the medium was replaced with fresh DMEM medium supplemented with 10% FBS.

The *C/EBP β* overexpression vector (pmCherry-N1-*C/EBP β*) was constructed using the pmCherry-N1 plasmid. KCs were planted in 12-well culture plates and transfected with 2 µg/ml pmCherry-N1 (empty vector) or 2 µg/ml pmCherry-N1-*C/EBP β* (C/EBP β) using lipofectamine 2000 (Invitrogen, USA). Six hours post transfection, the culture medium was changed to DMEM from Opti-MEM (Invitrogen, USA). Each transfection was independently repeated three times.

### RNA isolation and quantitative real-time PCR

Total RNAs were isolated from KCs using TRIzol reagents (Invitrogen, USA). Complementary DNA synthesis was performed using 1 µg total RNA via HiScriptII 1st Strand cDNA Synthesis Kit or miRNA 1st Strand cDNA Synthesis Kit (Vazyme, China). qRT-PCR was carried out using SYBR Green Premix Pro Taq HS qPCR Kit on ABI 7500 Thermal Cycler (Thermo, USA) with the following steps: 95 ℃ for 5 min and 40 cycles of 95 ℃ for 10 s, 60 ℃ for 30 s. The relative expression level was normalized to U6 small nuclear RNA (snRNA) or *GAPDH* using the 2^−ΔΔCt^ formula. The details of primers used for qRT-PCR are summarized in Table 1. Statistical analysis data were taken from three independent experiments.

**Table 1:** Primers for qRT-PCR

Primer	Sequence(5ʹ-3ʹ)
miR-374b-5p	GCGCGATATAATACAACCTGC
Universal reverse primer	GCTGTCAACGATACGCTACG
*C/EBP β* forward primer	GCTGAGCGACGAGTACAAGATGC
*C/EBP-β* reverse primer	CTTGTGCTGCGTCTCCAGGTTG
*IL6* forward primer	CTTCTTGGGACTGATGCTGGTGAC
*IL6* reverse primer	TCTGTTGGGAGTGGTATCCTCTGTG
*iNOS* forward primer	CCTAGTCAACTGCAAGAGAA
*iNOS* reverse primer	TTTCAGGTCACTTTGGTAGG
*TNFα* forward primer	CGCTCTTCTGTCTACTGAACTTCGG
*TNFα* reverse primer	GTGGTTTGTGAGTGTGAGGGTCTG
*Arg1* forward primer	AGACAGCAGAGGAGGTGAAGAGTAC
*Arg1* reverse primer	AAGGTAGTCAGTCCCTGGCTTATGG
*GAPDH* forward primer	CCACTCACGGCAAATTCAAC
*GAPDH* reverse primer	CTCCACGACATACTCAGCAC

### Target gene analyses of miR-374b-5p

miRDB (https://mirdb.org/) and TargetScan (https://www.targetscan.org/mmu_80/) were used to predict the targets of miR-374b-5p. According to the results, the potential target genes were selected for further validation.

### Plasmid construction and luciferase assay

The 3'-UTRs of *C/EBP β* were cloned into pmirGLO plasmid to construct the luciferase reporter plasmids also as pmirGLO-*C/EBP β*-WT. Meanwhile, the 3ʹ-UTRs of wild-type sequences of *C/EBP β* binding to seed region of miR-374b-5p were mutated and cloned into pmirGLO plasmid, named the pmirGLO-*C/EBP β*-Mut.

HEK293T cells (1 × 10^5^ cells per well) were plated into 24-well plates and co-transfected by pmirGLO-*C/EBP β*-WT (*C/EBP β*-WT) or pmirGLO-*C/EBP β*-Mut (*C/EBP β*-Mut) with miR-374b-5p mimics or miR-NC using Lipofectamine 2000 according to the manufacturer’s instructions, respectively. After 24 h, luciferase activity was detected on a Dual Glo Luciferase Assay System (Promega, USA) using a Dual Luciferase Reporter Assay System (Vazyme, China). For this assay, relative activity was defined by the ratio of firefly to Renilla luciferase activity. Each transfection was independently repeated three times.

### Western blot

First, cells were cleaned three times with pre-cooled PBS and lysed with RIPA buffer (Thermo, USA) containing protease-phosphatase inhibitor (NCM Biotech, China). The total protein concentration was measured using BCA kit (Vazyme, China). Total protein (25 µg) from each group was separated with 12% SDS-PAGE and then transferred to PVDF membranes. Subsequently, the PVDF membranes were blocked with 5% non-fat dried milk for 90 min at room temperature and washed once in TBS with 0.05% Tween (TBST). Then, the PVDF membranes were incubated with the following primary antibody overnight at 4 ℃: C/EBP β rabbit monoclonal antibody (1:1000, Beyotime, China), NF-κBp65 mouse monoclonal antibody (1:500, Beyotime, China), p-NF-κBp65 rabbit polyclonal antibody (1:500, Beyotime, China), rabbit anti-IL6 polyclonal antibody (1:1000, Bioss, China), rabbit anti-TNFα polyclonal antibody (1:500, Bioss, China), rabbit anti-iNOS polyclonal antibody (1:1000, Bioss, China), rabbit anti-Arg1 polyclonal antibody (1:1000, Bioss, China) and rabbit anti-β-actin polyclonal antibody (1:5000, Bioss, China). Then, rabbit anti-mouse IgG-HRP antibody (1:4000, Biodragon, China) or goat anti-rabbit IgG-HRP antibody (1:4000, Biodragon, China) was used to incubate with membranes for 1 h. Finally, the membranes were tested on a high-resolution image acquisition system (BioRad, USA) with BeyoECL Moon reagent (Beyotime, China).

### Statistical analysis

All data are shown as mean ± SD. The level of differential expression was evaluated between experimental group and control group by *t*-test. All the statistical analyses in this study were performed by GraphPad Prism 8. A *p* value < 0.05 was considered statistically significant.

## Results

### Increased expression of miR-374b-5p in KCs after *E. multilocularis* infection

**Fig. 1:**
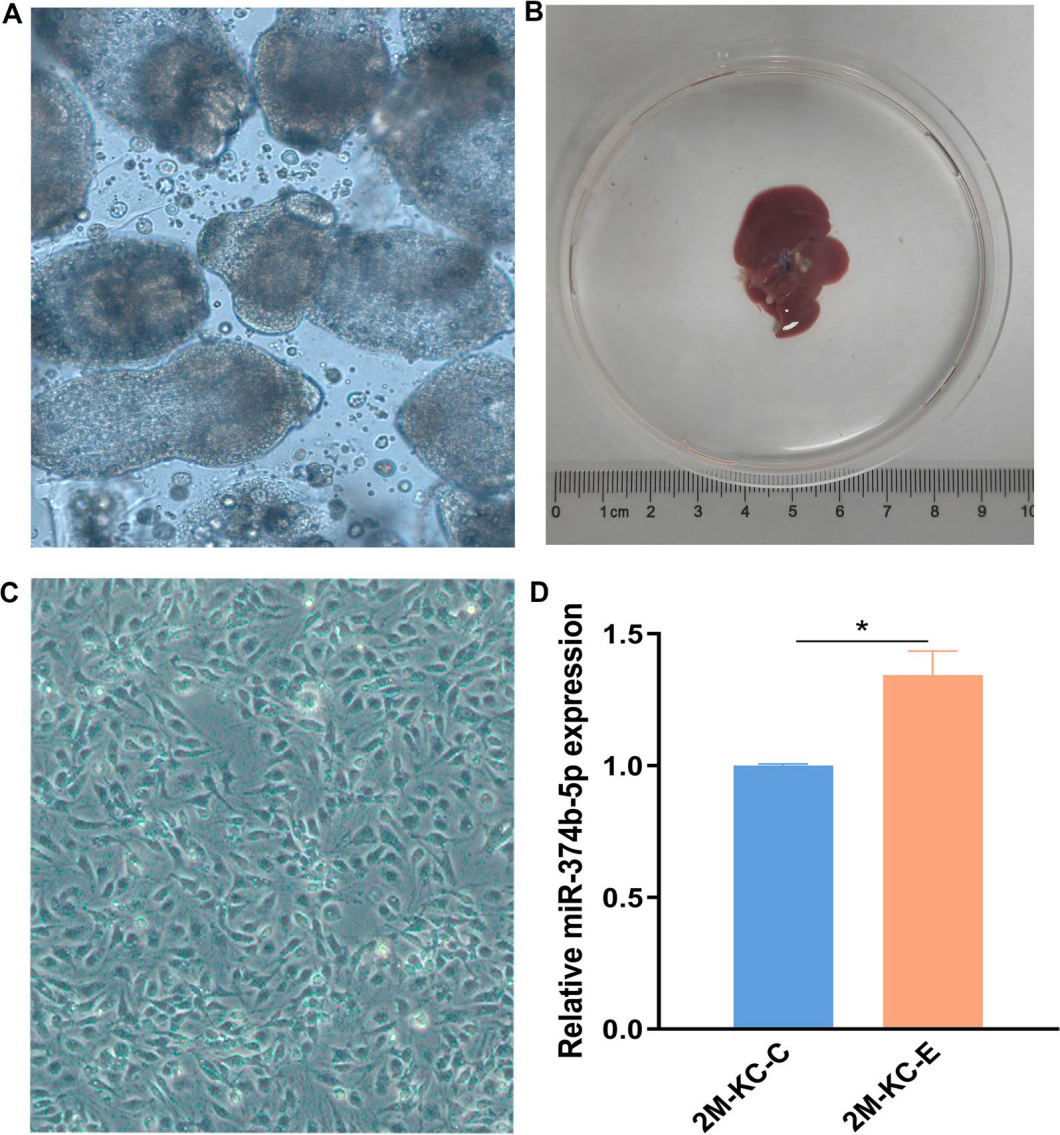
Expression level of miR-374b-5p in KCs at 2 months post-infection with *Echinococcus multilocularis*. **A** Morphology of *E. multilocularis* protoscolex was observed under a microscope (10 × 5). **B** Growth of liver cysts at 2 months post-infection. **C** Morphology of mouse KCs was observed under a microscope (10 × 20). **D** Expression level of miR-374b-5p in KCs at 2 months post-infection. The corresponding *p* value was calculated between experimental group and control group by *t*-test, *p < 0.05, **p < 0.01, ***p < 0.001. Abbreviations: 2 M-KC-C, KCs from mice treated with PBS for 2 months; 2 M-KC-E, KCs from mice 2 months post-infection with *E. multilocularis*

Protoscolices were collected from the *E. multilocularis*-infected Mongolian gerbil (Fig. 1A). Previous research has shown that miR-374b-5p is involved in pathogen-mediated inflammation [18, 19]. To examine the expression of miR-374b-5p in mouse KCs during *E. multilocularis* infection, the primary KCs were obtained from the liver of *E. multilocularis*-infected mice (Fig. 1B, C). The qRT-PCR result showed that the level of miR-374b-5p was significantly increased at 2 months post-infection (Fig. 1D).

### miR-374b-5p was directly bound to the 3'-UTR of C/EBP β and suppressed its expression

**Fig. 2:**
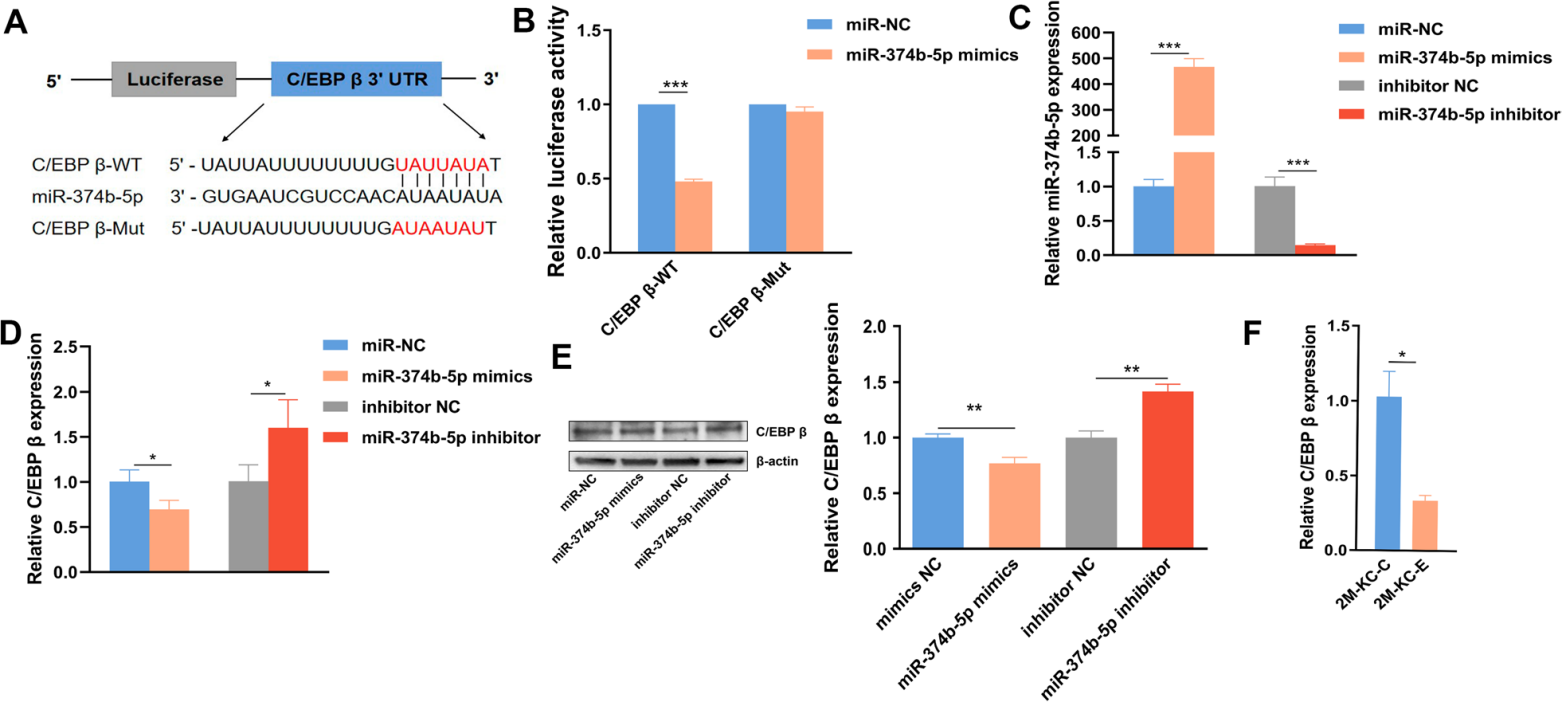
miR-374b-5p was directly bound to the 3ʹ-UTR of *C/EBP β* and suppressed its expression. **A** Putative binding site of miR-374b-5p in the 3ʹ-UTR of C/EBP β. **B** Relative luciferase activity in HEK293T cells co-transfected with miR-374b-5p mimics and pmirGLO-C/EBP β-WT or pmirGLO-C/EBP β-Mut, respectively. **C** Expression level of miR-374b-5p and (**D**) C/EBP β in KCs transfected with miR-374b-5p mimics, miR-374b-5p inhibitor or their NC. **E** Western blot analysis of C/EBP β expression in KCs transfected with miR-374b-5p mimics, miR-374b-5p inhibitor or their NC. **F** Expression level of C/EBP β in the KCs from *Echinococcus multilocularis*-infected mice. Corresponding *p* value was calculated between experimental group and control group by *t*-test, *p < 0.05,**p < 0.01, ***p < 0.001. C/EBP β-WT: pmirGLO-C/EBP β-Wildtype, C/EBP β-Mut: pmirGLO-C/EBP β-Mutant, 2 M-KC-C: KCs from mice treated with PBS for 2 months; 2 M-KC-E, KCs from mice 2 months post-infection with *E. multilocularis*

To determine the potential role of miR-374b-5p in KCs during *E. multilocularis* infection, its potential targets were predicted by TargetScan and miRDB databases. A total of eight potential targets, mainly associated with immune response, were screened out and visualized by qRT-PCR. Among these, only *C/EBP β* gene was significantly downregulated in miR-374b-5p-overexpressed KCs (Additional file 1: Fig. S1). Further analysis showed that there is a single binding site of miR-374b-5p in 3ʹ-UTR of *C/EBP β* (Fig. 2A). Luciferase reporter assay revealed that the miR-374b-5p significantly decreased the luciferase activity in the HEK293T cells transfected with pmirGLO-*C/EBP β*-WT compared with that in the NC control (Fig. 2B). However, the decrease was not observed in the HEK293T cells transfected with pmirGLO-*C/EBP β*-Mut (Fig. 2B), suggesting that miR-374b-5p can bind to the 3ʹ-UTR of *C/EBP β*. Furthermore, the expressions of C/EBP β were significantly inhibited at mRNA and protein levels in the miR-374b-5p-overexpressed KCs. Consistently, C/EBP β was significantly elevated at both mRNA and protein levels after downregulation of miR-374b-5p in the KCs by transfecting with miR-374b-5p inhibitor (Fig. 2C, D, E). Moreover, the expression of *C/EBP β* was negatively correlated with the miR-374b-5p expression in the KCs from *E. multilocularis*-infected mice (Fig. 2F). Above results suggest that miR-374b-5p was able to directly target to the 3ʹ-UTR of *C/EBP β* and thereby suppressed C/EBP β expression.

### Overexpression of miR-374b-5p inhibited the expression of NF-κBp65

**Fig. 3:**
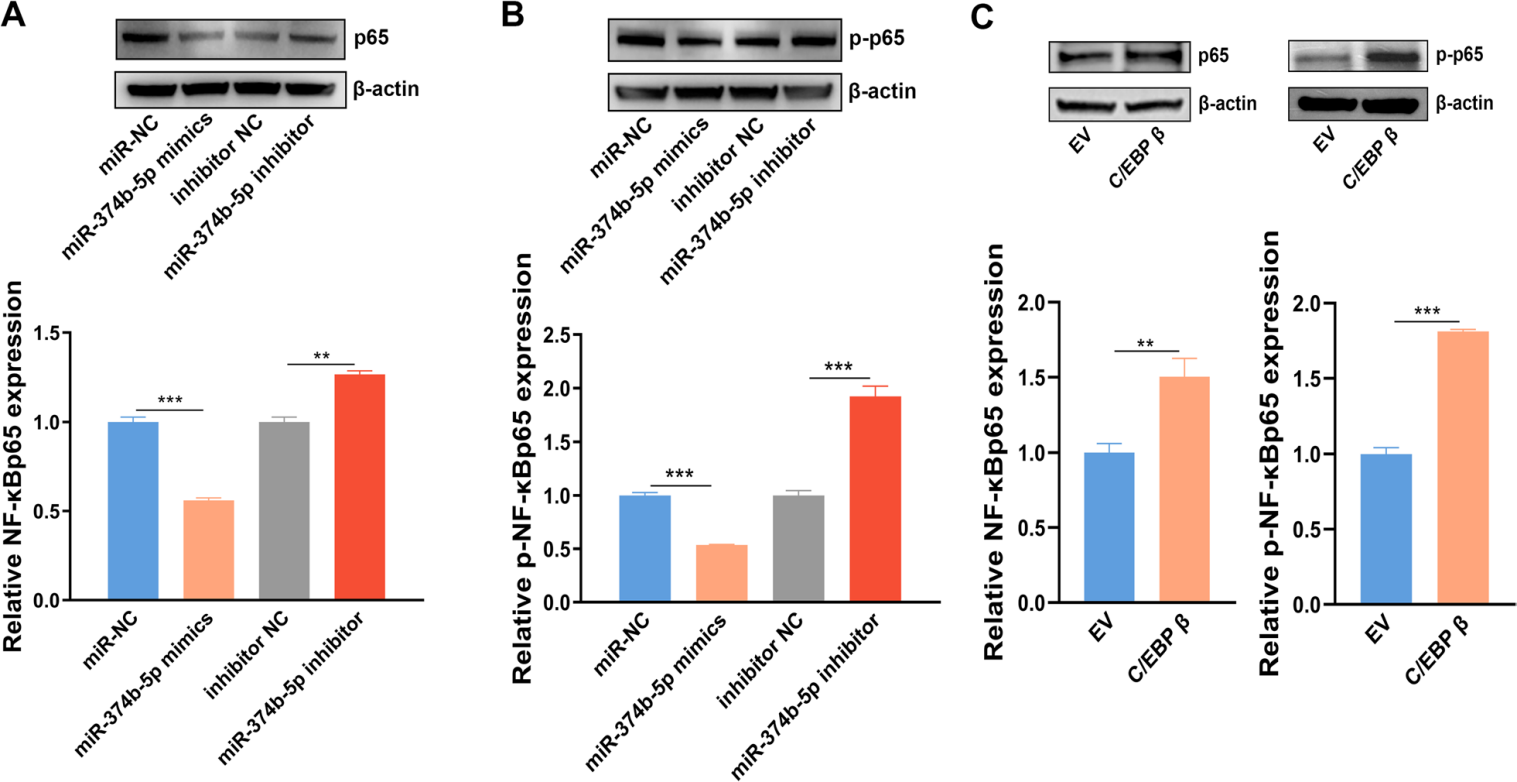
miR-374b-5p inhibited NF-κBp65 and p-NF-κBp65 by regulating C/EBP β expression. **A** Protein expression level of NF-κBp65 and (**B**) p-NF-κBp65 were detected by western blot in KCs transfected with miR-374b-5p mimics, miR-374b-5p inhibitor or NC. **C** Protein expression level of NF-κBp65 and p-NF-κBp65 was detected by western blot in KCs transfected with pmCherry-N1 empty vector or pmCherry-N1-C/EBP β. The corresponding *p* value was calculated between experimental group and control group by *t*-test, * *p* < 0.05, ** *p* < 0.01, *** *p* < 0.001. EV: empty vector, C/EBP β: pmCherry-N1-C/EBP β

To study the effect of miR-374b-5p-mediated C/EBP β on the NF-κB signaling pathway, the primary mice KCs were isolated. We found the protein levels of NF-κBp65 and p-NF-κBp65 were significantly downregulated in the KCs transfected with miR-374b-5p mimics, while their protein levels were significantly upregulated after transfection with miR-374b-5p inhibitor (Fig. 3A, B). Consistently, the protein levels of NF-κBp65 and p-NF-κBp65 were significantly upregulated in the KC overexpressed C/EBP β (Fig. 3C). This indicated that miR-374b-5p-mediated C/EBP β may be involved in the regulation of NF-κB signaling pathways.

### Overexpression of miR-374b-5p changed the level of inflammatory factors

**Fig. 4:**
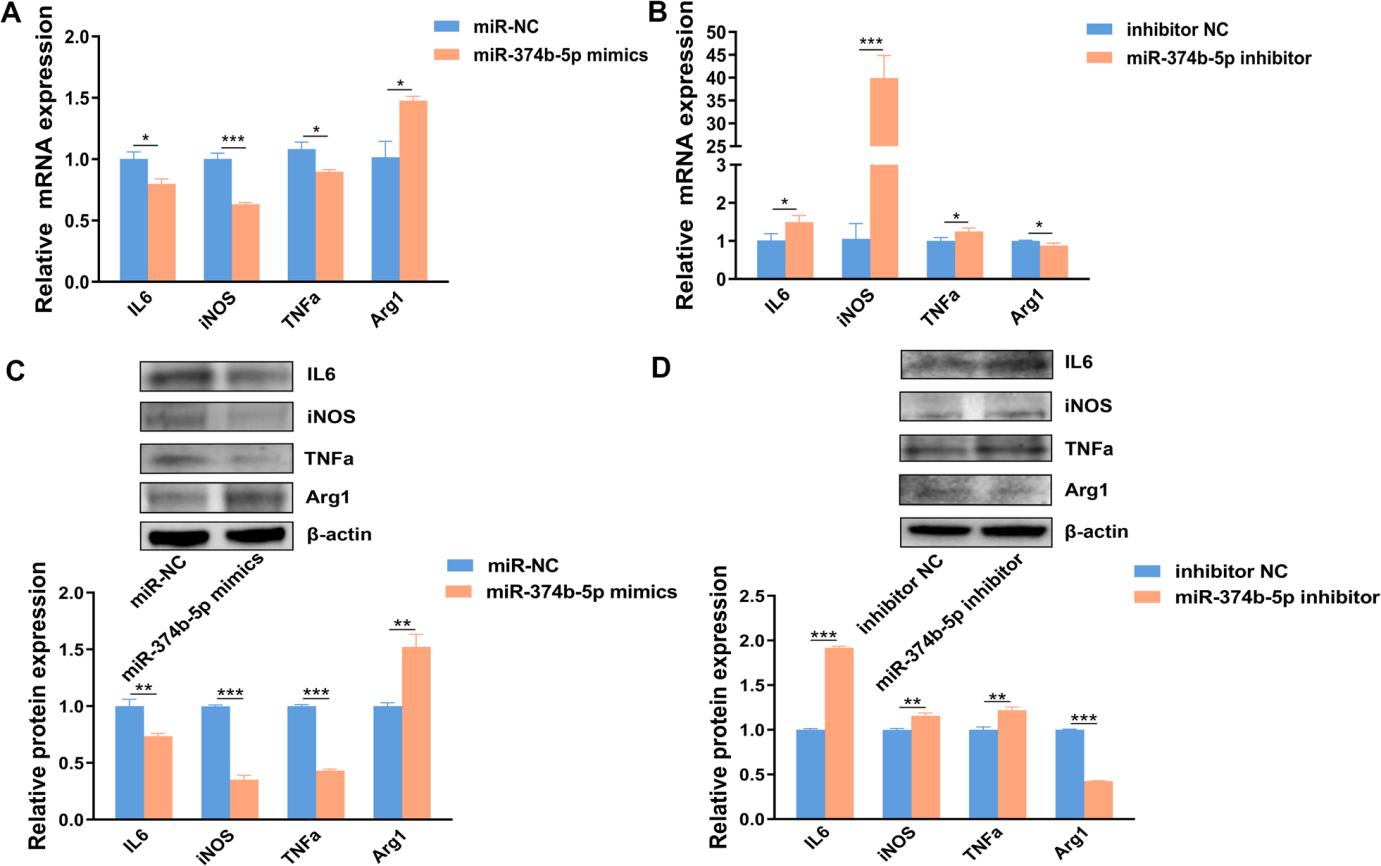
Effect of miR-374b-5p on inflammatory factor expression. **A** and **B** mRNA expression levels of pro-inflammatory factors and anti-inflammatory factors were detected by qRT-PCR in KCs transfected with miR-374b-5p mimics and miR-374b-5p inhibitor. **C** and **D** Expression levels of inflammatory factors were detected by western blot in KCs transfected with miR-374b-5p mimics and miR-374b-5p inhibitor. Corresponding* p* value was calculated between experimental group and control group by *t*-test, *p < 0.05,**p < 0.01, ***p < 0.001

To determine whether the miR-374b-5p can influence the expression of inflammatory factors, the expression levels of M1-type pro-inflammatory (iNOS, TNFα, and IL6) and M2-type anti-inflammatory factors (Arg1) were detected by qRT-PCR and western blot. The expressions of iNOS, TNFα and IL6 at both mRNA and protein levels were statistically downregulated in the KCs transfected with miR-374b-5p mimics compared with miR-NC, while Arg1 expression was statistically upregulated (Fig. 4A,C). After transfection with miR-374b-5p inhibitor, the expressions of those inflammatory factors were opposite to those in the miR-374b-5p-overexpressed KCs (Fig. 4B,D). Above all, miR-374b-5p probably induced M2-type anti-inflammatory factors while suppressed M1-type pro-inflammatory factors via C/EBP β/NF-κB signaling pathway.

### Production of proinflammatory factors was inhibited in KCs from *E. multilocularis*-infected mice and treated with ESPs

**Fig. 5:**
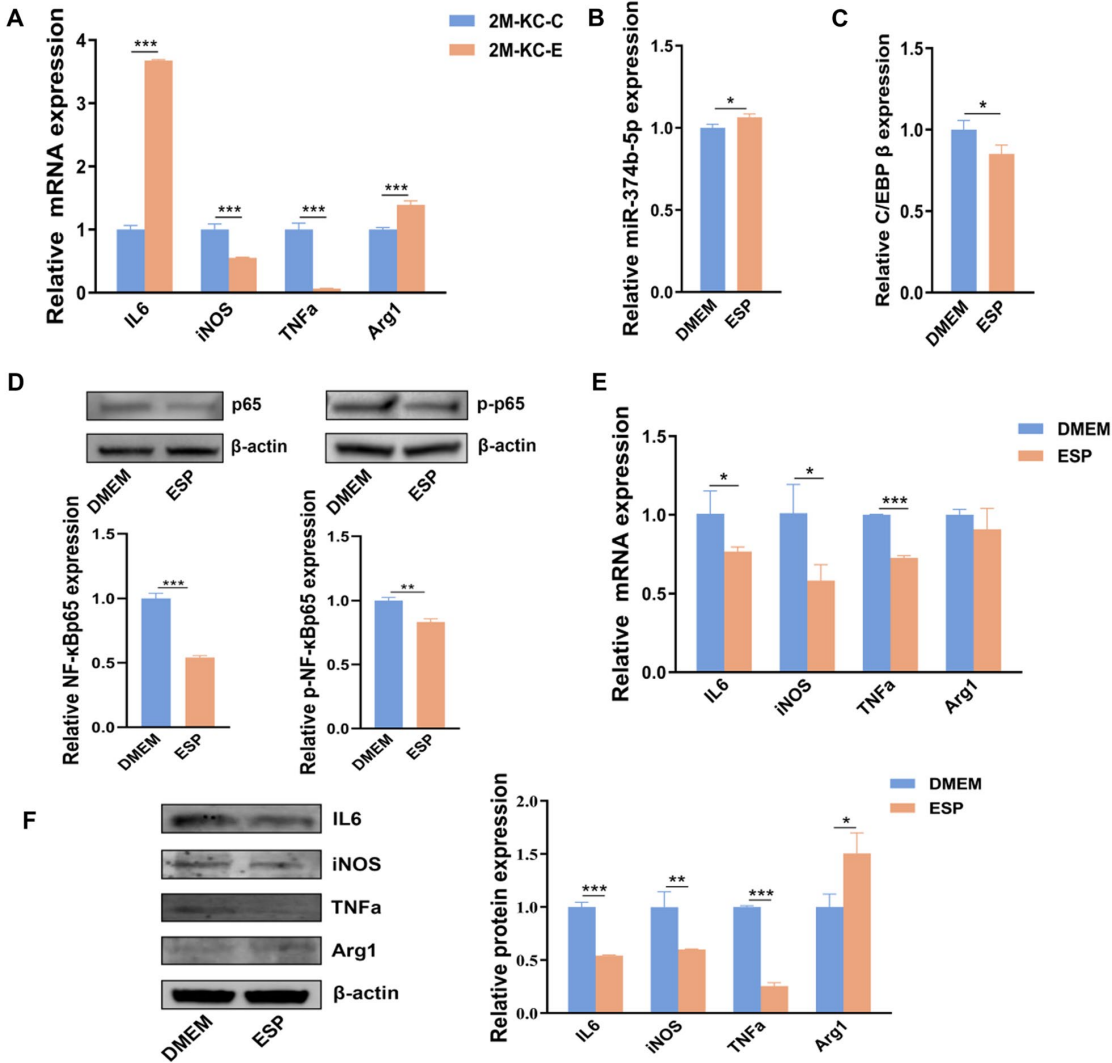
Production of proinflammatory factors was inhibited in KCs treated with ESPs. **A** mRNA expression levels of inflammatory factors in KCs from *Echinococcus multilocularis*-infected mice at 2 months post-infection. **B** mRNA expression levels of miR-374b-5p, (**C**) C/EBP β and (**E**) inflammatory factors in the ESP-treated KCs. **D** Protein expression levels of NF-κBp65 and p-NF-κBp65 were detected in the ESP-treated KCs. **F** Protein expression levels of inflammatory factors were detected in the ESP-treated KCs. Corresponding* p* value was calculated between experimental group and control group by *t*-test, *p < 0.05, **p < 0.01, ***p < 0.001. 2 M-KC-C: KCs from mice treated with PBS for 2 months, 2 M-KC-E: KCs from mice 2 months post-infection with *E. multilocularis*, ESP: excretory/secretory products

The results showed that the expression levels of *iNOS* and *TNFα* were decreased in KCs at 2 months post-infection, while *Arg1* was obviously increased (Fig. 5A). Interestingly, the expression of miR-374b-5p was upregulated in KCs treated with 25 µg ESPs (Fig. 5B), and its target genes *C/EBP β* were downregulated (Fig. 5C). Furthermore, the protein expressions of NF-κBp65 and p-NF-κBp65 were downregulated in ESP-treated KCs (Fig. 5D). The expressions of iNOS, TNFα and IL6 at mRNA and protein levels were statistically downregulated in KCs treated with ESPs. The mRNA level of *Arg1* had no change after treatment with ESPs, but *Arg1* protein was statistically upregulated (Fig. 5E, F). All these findings demonstrated that the NF-κB signaling was inhibited after treating KCs with ESPs in vitro.

## Discussion

Alveolar echinococcosis (AE) is an important infectious disease, threatening the health of human beings and animals worldwide. How to effectively prevent and treat the AE is still a challenge. *Echinococcus multilocularis* causes continuous and infiltrative tumor-like invasive growth in liver, leading to serious liver injury. Recent research shows that KCs are a major source of inflammatory mediators [22] and closely related to the immune response in liver. KCs located in the hepatic sinusoid can play a defensive role by releasing inflammatory mediators. Previous study showed that miRNA profiles of KCs change significantly during *E. multilocularis* infection [20]. In this study, we found that miR-374b-5p was upregulated at the middle stage of infection. Evidence showed that miR-374b-5p was involved in the regulation of inflammation response. It was reported that overexpression of miR-374b-5p can lead to the reduction of neuroinflammation in Alzheimer's disease models [23]. Previous study showed the immune regulatory effect of miR-374b-5p on CD4^+^ T cells on mouse spleen [19].

Luciferase reporter assay revealed that the miR-374b-5p could directly bind to C/EBP β. Moreover, downregulation of C/EBP β in the KCs transfected with miR-374b-5p mimics was observed. C/EBP β, one of the transcription factors, can combine with the promoter and thus control the expression of target genes [24]. Studies found that C/EBP β is closely related to the NF-κB signaling pathway; C/EBP β promotes NF-κBp65 translocation into the nucleus and DNA binding activity [25, 26]. The result showed that overexpression of miR-374b-5p inhibited the protein levels of NF-κBp65 and p-NF-κBp65. Consistently, overexpression of C/EBP β promoted the protein levels of p-NF-κBp65. NF-κBp65 was discovered as the critical protein responsible for NF-κB signaling pathway. When NF-κBp65 subunit is phosphorylated and transferred into the nucleus from the cytoplasm [27, 28], the phosphorylation of NF-κBp65 results in NF-κB activation and production of proinflammatory cytokines [29, 30]. Experimental results also showed that overexpression of miR-374b-5p inhibited the production of pro-inflammatory factors while promoting the expression of anti-inflammatory factors in primary mouse KCs. Moreover, the production of proinflammatory factors was inhibited in KCs from *E. multilocularis*-infected mice. This result indicated that miR-374b-5p is probably involved in the inflammatory reaction process during the *E. multilocularis* infection. It has been confirmed that the parasitic helminths stimulate the host to trigger the Th1-type immune response in early stage infection [31]. Subsequently, Th1-type immune response gradually changes to the Th2-type immune response, which contributes to survival of the worms in the host. Some researchers [32, 33] found that, in the early stage of worm infection, anti-inflammatory factors showed an upward trend. Our research results also support this finding. Helminths can manipulate the host immune response toward Th2-type immune response, which is mainly produced in response to both the mechanical extrusion caused by the helminths and their release of excretory/secretory products [34, 35]. To confirm these changes are due to *E. multilocularis* infections, ESPs were collected and co-cultured with KCs. As expected, miR-374b-5p was obviously upregulated in KCs treated with ESPs, consistent with KCs from infested *E. multilocularis*. The protein expressions of NF-κBp65 and p-NF-κBp65 were downregulated in ESP-treated KCs. Then, the inflammatory cytokines in KCs treated with ESPs were tested. The result reflects that the expressions of iNOS, TNFα and IL6 at mRNA and protein levels were statistically downregulated in KCs treated with ESPs. These results were similar to those seen in miR-374b-5p-overexpressed KCs. The mRNA level of *IL6* was upregulated at 2 months post-infection in KCs, possibly because of the very complex regulatory mechanism of animals. Several recent studies have shown that IL6 not only promotes M2 polarization but also suppresses the production of proinflammatory cytokines [36–38].

## Conclusions

Our results indicate that miR-374b-5p was obviously upregulated at 2 months post-infection in KCs. Moreover, miR-374b-5p was able to bind to the 3ʹ-UTR of the *C/EBP β* gene and suppress its expression, thus downregulating NF-κB signaling. This research is helpful to explore the mechanism of inflammation regulation and host defense during *E. multilocularis* infection.

### Supplementary Information

**Additional file 1: Figure S1.** mRNA expression levels of potential targets in KCs transfected with miR-374b-5p mimics were detected by qRT-PCR. **Table S1.** Putative target genes of mmu-miR-374b-5p.

## Data Availability

The datasets supporting the findings of this article are included within the article.

## Abbreviations

*E. multilocularis*: *Echinococcus* *multilocularis*
AE: Alveolar echinococcosis
miRNA: MicroRNA
KCs: Kupffer cells
FBS: Fatal bovine serum
3 'UTR: 3 'Untranslated region
WT: Wild type
Mut: Mutant
PBS: Phosphate-buffered solution
SDS-PAGE: Sodium dodecyl sulfate-polyacrylamide gel electrophoresis
TBST: Tris-buffered saline with Tween-20
PVDF: Polyvinylidene fluoride
iNOS: Induced nitrogen monoxide synthase
TNFα: Tumor necrosis factor α
IL6: Interleukin 6
Arg1: Arginase1
