# Supplementary material for: mmu-miR-374b-5p modulated inflammatory factors via downregulation of C/EBP β/NF-κB signaling in Kupffer cells during Echinococcus multilocularis infection
Source: Parasit Vectors. 2024 Mar 29;17:163. doi: 10.1186/s13071-024-06238-0 (PMC10981327; doi:10.1186/s13071-024-06238-0)
Supplement: Supplementary file 1 — Additional file 1: Figure S1. mRNA expression levels of potential targets in KCs transfected with miR-374b-5p mimics were detected by qRT-PCR. Table S1. Putative target genes of mmu-miR-374b-5p. [file 13071_2024_6238_MOESM1_ESM.docx]

Supplementary Material

**1 Supplementary Figure and Table**

**1.1 Supplementary Figure**

**
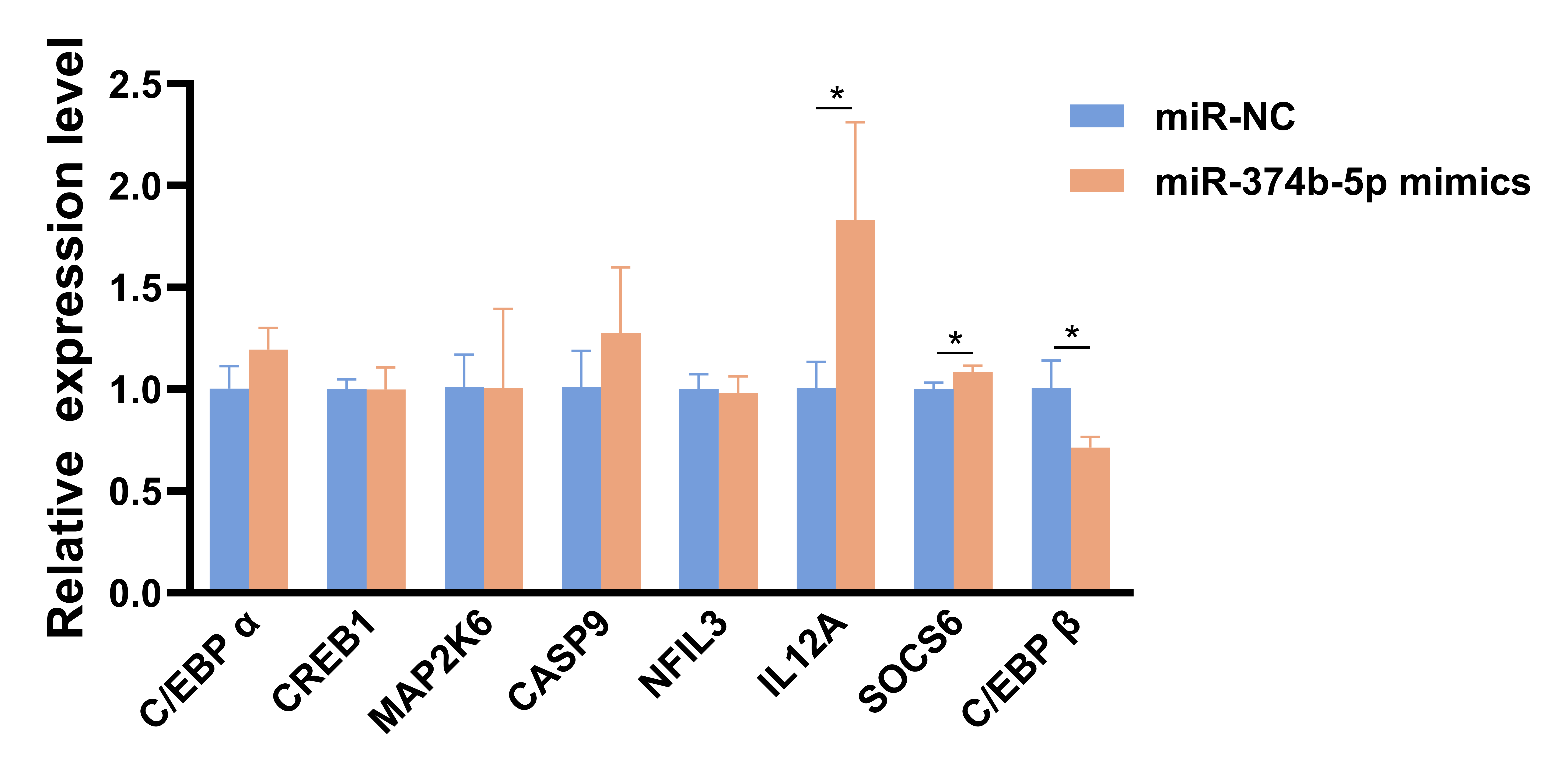
**

**Figure S1 The mRNA expression levels of potential targets in KCs transfected with miR-374b-5p mimics were detected by qRT-PCR.**

**1.2 Supplementary Table**

**Table S1** **Putative target genes of mmu-miR-374b-5p**

| **Gene name** | **Gene ID** | **Gene function** | **Reference** |  |
| --- | --- | --- | --- | --- |
| *C/EBP α* | 12606 | Induce the formation of functional macrophages | (Laiosa et al., 2006) | |
| *CREB1* | 12912 | Regulate Creb1/Klf4 signaling pathway | (Ou et al., 2021) | |
| *MAP2K6* | 26399 | Regulate MAPK signaling pathway | (Bouraoui et al., 2018) | |
| *CASP9* | 12371 | Mediate inflammation | (Colón et al., 2022) | |
| *NFIL3* | 4783 | Regulate NF-κB signaling pathway | (Yang et al., 2022) | |
| *IL12A* | 16159 | Regulate Macrophage Polarization | (Wang et al., 2021) | |
| *SOCS6* | 54607 | Negative regulators of cytokine receptor signaling | (Kabir et al., 2014) | |
| *C/EBP β* | 12608 | Regulate NF-κB signaling pathway | (Yang et al., 2017) | |

[1] Laiosa CV, Stadtfeld M, Xie H, de Andres-Aguayo L, Graf T. Reprogramming of committed T cell progenitors to macrophages and dendritic cells by C/EBP alpha and PU.1 transcription factors. Immunity. 2006 Nov;25(5):731-44. doi: 10.1016/j.immuni.2006.09.011. PMID: 17088084.

[2] Ou DL, Chen CW, Hsu CL, Chung CH, Feng ZR, Lee BS, Cheng AL, Yang MH, Hsu C. Regorafenib enhances antitumor immunity via inhibition of p38 kinase/Creb1/Klf4 axis in tumor-associated macrophages. J Immunother Cancer. 2021 Mar;9(3):e001657. doi: 10.1136/jitc-2020-001657. PMID: 33753566; PMCID: PMC7986673.

[3] Bouraoui Y, Achour M, Royuela M, Oueslati R. Immune profiling of human prostate epithelial cells determined by expression of p38/TRAF-6/ERK MAP kinases pathways. Kaohsiung J Med Sci. 2018 Mar;34(3):125-133. doi: 10.1016/j.kjms.2017.10.002. Epub 2017 Oct 26. PMID: 29475459.

[4] Colón Ortiz C, Neal AM, Avrutsky MI, Choi M, Smart J, Lawson J, Troy CM. Neurovascular injury associated non-apoptotic endothelial caspase-9 and astroglial caspase-9 mediate inflammation and contrast sensitivity decline. Cell Death Dis. 2022 Nov 8;13(11):937. doi: 10.1038/s41419-022-05387-3. Erratum in: Cell Death Dis. 2022 Nov 24;13(11):993. PMID: 36347836; PMCID: PMC9643361.

[5] Yang W, Li J, Zhang M, Yu H, Zhuang Y, Zhao L, Ren L, Gong J, Bi H, Zeng L, Xue Y, Yang J, Zhao Y, Wang S, Gao S, Fu Z, Li D, Zhang J, Wang T, Shan M, Tang B, Li X. Elevated expression of the rhythm gene NFIL3 promotes the progression of TNBC by activating NF-κB signaling through suppression of NFKBIA transcription. J Exp Clin Cancer Res. 2022 Feb 18;41(1):67. doi: 10.1186/s13046-022-02260-1. PMID: 35180863; PMCID: PMC8855542.

[6] Wang Z, Liu M, Ye D, Ye J, Wang M, Liu J, Xu Y, Zhang J, Zhao M, Feng Y, Xu S, Pan W, Luo Z, Li D, Wan J. Il12a Deletion Aggravates Sepsis-Induced Cardiac Dysfunction by Regulating Macrophage Polarization. Front Pharmacol. 2021 Jul 2;12:632912. doi: 10.3389/fphar.2021.632912. PMID: 34276358; PMCID: PMC8284189.

[7] Kabir NN, Sun J, Rönnstrand L, Kazi JU. SOCS6 is a selective suppressor of receptor tyrosine kinase signaling. Tumour Biol. 2014 Nov;35(11):10581-9. doi: 10.1007/s13277-014-2542-4. Epub 2014 Aug 30. PMID: 25172101.

[8] Yang X, Zou J, Cai H, Huang X, Yang X, Guo D, Cao Y. Ginsenoside Rg3 inhibits colorectal tumor growth via down-regulation of C/EBPβ/NF-κB signaling. Biomed Pharmacother. 2017 Dec;96:1240-1245. doi: 10.1016/j.biopha.2017.11.092. Epub 2017 Nov 21. PMID: 29169725.
